# Decision-Making in the Surgical Treatment of Breast Cancer: Factors Influencing Women’s Choices for Mastectomy and Breast Conserving Surgery

**DOI:** 10.3389/fonc.2016.00074

**Published:** 2016-03-29

**Authors:** Emily Catherine Bellavance, Susan Beth Kesmodel

**Affiliations:** ^1^Department of General and Oncologic Surgery, University of Maryland, Baltimore, MD, USA

**Keywords:** mastectomy, breast cancer, breast conserving surgery, shared decision-making, contralateral prophylactic mastectomy

## Abstract

One of the most difficult decisions a woman can be faced with when choosing breast cancer treatment is whether or not to undergo breast conserving surgery or mastectomy. The factors that influence these treatment decisions are complex and involve issues regarding access to health care, concerns for cancer recurrence, and the impact of surgery on body image and sexuality. Understanding these factors will help practitioners to improve patient education and to better guide patients through this decision-making process. Although significant scientific and societal advances have been made in improving women’s choices for the breast cancer treatment, there are still deficits in the decision-making processes surrounding the surgical treatment of breast cancer. Further research is needed to define optimal patient education and shared decision-making practices in this area.

## Introduction

Medical decision-making has evolved over the last several decades from one based on paternalism, in which the physician decided on the best course of treatment according to his/her view of what was in the best interest of the patient, to one focused on patient autonomy, in which the informed patient makes decisions about accepting or declining treatment options based on his/her own values and priorities. In modern medical ethics, shared decision-making has been proposed as the ideal model for medical decision-making that both acknowledges patient autonomy and the role of the physician in providing expert medial opinion. Shared decision-making is a process that informs patients about what available treatments are most effective under particular circumstances, incorporates patients’ needs and values into decisions, and improves the patient–clinician dialog about decisions (1, 2). Shared decision-making has been advocated as an ideal model to address treatment decisions in which no single treatment option is clearly indicated above others based on available medical evidence (2). Therefore, this model is particularly suited to treatment decisions in the management of the primary tumor in breast cancer, as a patient may face several surgical treatment options that result in equivalent oncologic outcomes.

One of the most difficult decisions a woman can be faced with when choosing breast cancer treatment is whether or not to undergo breast conserving surgery (BCS) or mastectomy. Notably, the clinical research in breast cancer treatment, which supports the use of BCS, came about at the time when women were also becoming dissatisfied with the traditional paternalistic doctor–patient relationship model and were demanding to have more choice in their medical care. In his book, “The Breast Cancer Wars,” Barron Lerner chronicles the convergence of the women’s liberation movement and the rise of BCS as the standard of surgical care in the treatment of early stage disease (3). In 1971, the writer Babette Rosamond was diagnosed with breast cancer when one of the first proponents of BCS, Bernard Crile, was offering a partial mastectomy as opposed to the traditional one-step procedure in which a woman with a suspicious breast mass was consented for an excisional biopsy under anesthesia and if this mass was determined to be a cancer on frozen section, the surgeon would then proceed with a radical mastectomy, which included the removal of the breast, overlying skin, axillary lymph nodes, and pectoralis muscles. When Babette Rosamond was presented with the one-step procedure, she refused and only gave permission for the excisional biopsy. The excision demonstrated a small focus of breast cancer. She then refused the radical mastectomy and sought out the opinion of Dr. Crile at the Cleveland Clinic who cited data from retrospective studies of less aggressive surgery, resulting in acceptable outcomes. Ms. Rosamond wrote about her experience in an article, “The Right to Choose,” in the popular woman’s journal McCall’s Magazine and ultimately published a book entitled, “The Invisible Worm.” She joined a host of other women leaders of the time who were vocal proponents against the current medical establishment’s support of aggressive breast cancer surgery who demanded choice in their cancer care and the option of less aggressive and more cosmetic procedures.

Concurrently, in the late 1960s, the surgeon Bernard Fisher developed and promoted a biological model of breast cancer in which he proposed that breast cancer was a systemic disease requiring both local and systemic treatment (4). Therefore, more radical surgery was not necessarily beneficial in the face of disseminated tumor cells. Although commending Crile and others for pursuing BCS, Fisher demanded more rigorous evidence to support less aggressive surgery by means of randomized clinical trials. Under his leadership, the National Surgical Adjuvant Breast and Bowel Project (NSABP) B04 and B06 trials were conducted. The NSABP-B04 trial demonstrated that sparing the pectoralis muscles in mastectomy did not negatively affect oncologic outcomes (5). The NSABP-B06 trial established that BCS results in equivalent overall survival as mastectomy in patients with early stage breast cancer (6). The addition of adjuvant radiation treatment to BCS decreased the rate of local recurrence from 39 to 14% over 20 years. To date, there are multiple randomized clinical trials with long-term follow-up demonstrating no difference in overall survival between BCS with adjuvant radiation and mastectomy for the treatment of operable breast cancers (6–11). Refinement of radiation techniques and the addition of adjuvant systemic therapies have further decreased the rate of local recurrence in BCS to approach that of mastectomy (12, 13). Currently, one of the quality assurance standards for the National Accreditation Program for Breast Centers in the United States is that at least 50% of Stages 1–2 breast cancers amenable to BCS are treated with partial mastectomy.

## Mastectomy and Breast Conservation

Despite data supporting BCS in eligible patients, a significant percentage of women who would be candidates for BCS still decide to undergo mastectomy. The factors that influence these treatment decisions are complex and involve issues regarding access to health care, concerns for cancer recurrence, and the impact of surgery on body image and sexuality. Understanding these factors will help practitioners to improve patient education and to better guide patients through this decision-making process.

Access to health care is one of the major determinants of choice for breast cancer surgery, especially with regard to access to specialty providers and treatment facilities. Because adjuvant radiation therapy is usually recommended after BCS, multiple studies have focused on the availability of radiation oncology specialists. A recent publication using data from the surveillance, epidemiology, and end results (SEER) database and the Health Resources and Services Administration Area Resource File evaluated the association between the choice of breast surgery (mastectomy or BCS), the receipt of adjuvant radiation therapy after BCS, and the density of radiation oncologists in a particular area (ROD) (14). The study demonstrated that the likelihood of a woman undergoing BCS for early stage breast cancer increased as the ROD in an area increased. In addition, the likelihood that adjuvant radiation therapy was omitted after BCS decreased as the ROD in an area increased. The results from this study are consistent with those of a large study using the Medicare database that evaluated the use of BCS in older breast cancer patients and demonstrated that BCS was used more frequently in counties with a high density of radiation oncologists (15).

Numerous studies have also demonstrated that travel distance for radiation therapy may be associated with decisions regarding BCS and the actual delivery of adjuvant radiation therapy after BCS (16–21). The largest of these studies evaluated the use of BCS in women with early stage breast cancer using the SEER database (17). This study showed that the use of BCS was more common when women received treatment in a hospital with a radiation facility compared to women living a greater distance from a hospital with a radiation center. This was statistically significant for women who resided ≥15 miles from the nearest hospital with a radiation treatment center (OR 0.52, 95% CI 0.46–0.58). The study also demonstrated that for women who had BCS, a statistically significant decrease in the use of adjuvant radiation therapy was observed in patients who lived ≥40 miles from a hospital with a radiation facility, although this only accounted for 1.7% of the patients in the study. The use of accelerated radiation schedules, including shorter course whole breast irradiation given over 3 weeks and partial breast irradiation, may help to ameliorate some of these issues by providing patients with more manageable radiation schedules.

The use of multidisciplinary treatment teams is becoming more common in the management of breast cancer patients, especially at larger, academic institutions where breast cancer specialists are available in multiple disciplines. However, a significant percentage of patients still do not have the opportunity to meet with a medical oncologist or radiation oncologist before undergoing surgery for breast cancer. One of the benefits of a multidisciplinary approach is that patients understand all the components of their breast cancer treatment prior to starting treatment, and this increased knowledge may have an impact on treatment decisions regarding surgery for breast cancer. In a study of elderly women aged ≥65 years with local or regional breast cancer treated from 1994 to 1995, those patients who had a consultation with a radiation oncologist preoperatively were 6.7 times more likely to have BCS compared to those who did not (*P* ≤ 0.001). Furthermore, the odds of a patient receiving adjuvant radiation therapy after BCS were five times greater for patients who had a preoperative radiation oncology consultation (*P* < 0.001). Although this study was conducted at the time when multidisciplinary care was not as prevalent as it is today, it did demonstrate how multidisciplinary care may influence treatment choices (22). Several studies have demonstrated that surgeon characteristics including practice setting and gender have an impact on BCS rates (23–25). Surgeons who are affiliated with academic institutions, whether or not they have fellowship training in breast surgery or surgical oncology, use BCS more often than community surgeons (23). This may be due to the greater availability of other specialty providers at academic institutions and the use of multidisciplinary care in this setting. The number and availability of reconstructive surgeons at a particular institution have also been shown to impact rates of mastectomy and reconstruction and BCS (19). In an analysis of patients treated for breast cancer at National Comprehensive Cancer Network institutions, a greater number of reconstructive surgeons were associated with increased mastectomy and reconstruction rates, whereas long wait times for breast reconstructive surgery were associated with increased BCS rates.

Factors predicting the use of BCS, including clinicopathologic, socioeconomic, and patient characteristics, have been examined in numerous studies. Tumor characteristics, including tumor size, lymph node involvement, and stage, have all been shown to influence treatment decisions, with BCS used more frequently in patients with smaller tumors (23, 26) without lymph node involvement (15) and mastectomy used more often in patients with higher stage (27). Socioeconomic factors, including higher education, low poverty areas, and private insurance, are also associated with increased use of BCS (15, 21, 24). Significant geographic variation also exists in the use of BCS, both local and regional. Multiple studies have demonstrated that patients living in the Northeast and Pacific West are more likely to have BCS than those in the South (15, 23, 26, 28). In an analysis of older breast cancer patients, 70% of the patients in the Northeast had BCS compared to 48–50% of patients in the South (*P* < 0.001) (15). In this study, patients in metropolitan areas were also more likely to have BCS than patients in rural areas. This may simply reflect decreased access to health care and particularly breast cancer specialists. This geographic variation may also be influenced by other factors, including education and socioeconomic status.

Although some single institution studies have shown that younger patient age is associated with the use of BCS (15, 21, 23, 24), more recent analysis of large national databases suggest that this trend has reversed. Two reviews of the National Cancer Database have demonstrated in the setting of an overall increase of BCS, younger patients are being treated with mastectomy at higher rates than their older counterparts after adjusting for patient, facility, and tumor characteristics (28, 29). The subset of women aged ≤35 years was twice as likely to undergo mastectomy compared to women aged 61–64 years (29). These studies also reported similar trends with socioeconomic status, geography, and cancer stage outlined above, with a more recent narrowing of the BCS disparity in the South (28). In addition, access to radiation also appeared to influence BCS rates in these studies. It is unclear why younger women may be opting for more extensive surgery. This may be due to a concern for locoregional recurrence in younger patients (30), although more aggressive surgery does not appear to affect breast cancer-specific survival (31). Increased awareness of familial breast cancer syndromes may also be affecting mastectomy rates in younger women, who are at higher risk for having a deleterious genetic mutation and therefore may be choosing bilateral mastectomy for the treatment of a unilateral cancer.

When patients are diagnosed with breast cancer, they obtain support and advice from multiple sources when making decisions regarding breast surgery. The surgeon’s recommendation or preference for care is frequently cited as an important factor in this decision-making process. In a survey study that examined breast cancer care in a group of 96 patients, women who chose BCS indicated that the most important factor in the decision was the surgeon (32). This was in contrast to patients who selected mastectomy with or without reconstruction, where fear of cancer and concern about radiation therapy were ranked as more significant factors.

One of the major goals for providers is to help patients make informed decisions about their care. The development and use of decision-making aids have been investigated by several groups as a way to help providers obtain a better understanding of patient preferences for treatment (33, 34). These aids may also enhance patient decision-making by improving delivery of information and facilitating communication between providers and patients. In one study, patients and surgeons were interviewed to identify key factors influencing breast cancer surgery decisions, which were then incorporated into a decision board that could be reviewed at the time of surgical consultation (34). For patients, information on options for reconstruction, quality of life, and body image was important factors, whereas for surgeons, details regarding treatment side effects were considered important. The decision board was administered to 175 patients and 98% reported that it was easy to understand and 81% indicated that it helped in the decision process. Surgeons also found the decision board to be helpful in presenting information to patients. A subsequent randomized trial comparing the decision board to usual care demonstrated that patients who had surgical consultations with the decision board had higher knowledge scores regarding treatment options (66.9 vs. 58.7, *P* < 0.0001), less decisional conflict (1.40 vs. 1.62, *P* = 0.02), and were more satisfied with the decision-making process (4.50 vs. 4.32, *P* = 0.05). In addition, patients in the decision board group were more likely to undergo BCS (94 vs. 76%, *P* = 0.03). A similar approach using an interactive CD-ROM decision aid showed that patients using the CD-ROM were more satisfied with the amount of information received, their treatment decisions, and the decision-making process (33). However, the CD-ROM decision aid had no impact on treatment decisions. A recent meta-analysis of studies using decision aids in breast cancer patients, which included the above studies, demonstrated that in the three randomized trials of decision aids, women were 25% more likely to choose BCS over mastectomy if a decision aid was utilized (risk ratio 1.25, 95% CI 1.11–1.40) (35). In addition, decision aids increased patient knowledge by 24%, decreased decisional conflict, and improved the overall decision-making process.

## Body Image and Breast Reconstruction

An important concern for women undergoing breast cancer surgery is the impact this will have on body image and sexuality. Some studies have demonstrated that women undergoing BCS have fewer concerns about body image compared to mastectomy patients (36–39), whereas others have found no difference between the BCS and mastectomy groups (40, 41). In a recent meta-analysis of 12 studies on body image after breast cancer surgery, Fang et al. demonstrated that BCS patients had a better overall body image than women undergoing mastectomy with reconstruction and scored higher on body stigma domain (42). However, reconstruction significantly improved body image in mastectomy patients compared to no reconstruction. In addition, cosmetic satisfaction in postmastectomy patients with reconstruction appears to be high (43, 44). Currently, in the United States, universal coverage for postmastectomy reconstruction is mandated based on the passing of the Women’s Health and Cancer Rights Act in 1998. Despite the majority of patients do not undergo reconstruction (19). Factors associated with not receiving postmastectomy reconstruction include social and racial disparities, including black race, lower educational level and income, and public insurance (45–48). Although the racial disparity with breast reconstruction has been shown in multiple studies, a review of the Department of Defense cancer database shows that the receipt of reconstruction between White and Black women was equivalent, suggesting that the racial disparity with reconstruction may not be as evident when access is equal (49). Other factors associated with low reconstruction rates include older patient age, advanced disease, presence of comorbidities, and lack of access to reconstructive surgeons (19, 45–47). Although exogenous factors influencing reconstruction rates can be identified by institutional and database reviews, few studies have examined patients’ perspective of decision-making about breast reconstruction. In a survey study of breast cancer patients sampled from the SEER database, the majority of mastectomy patients reported satisfaction with the decision-making process about reconstruction. Dissatisfaction was associated with race, with black and Latina women being less satisfied, but was not associated with income or educational level. The most common reasons cited by patients for not undergoing reconstructive surgery are to avoid additional surgery and that they did not feel reconstruction was important. The main systems barrier reported to obtain reconstruction was lack of insurance coverage, whereas knowledge of the reconstruction as an option and finding a reconstructive surgeon were not significant barriers (45).

## Contralateral Prophylactic Mastectomy

Contralateral prophylactic mastectomy (CPM) is the removal of the healthy breast in the treatment of a unilateral cancer. Reviews of large national databases in the United States have demonstrated an increase in the rates of CPM in cases of operable breast cancer by over 150% (50, 51). This trend has also been reproduced in multiple single institution studies, with centers reporting CPM rates as high as 24% in the treatment of mastectomy patients (52, 53). These data are notable for the finding that patient factors are often more powerful predictors than tumor factors. Specifically, White race, higher socioeconomic status, and young age have been consistently identified as independent predictors for CPM (50, 52, 53). Despite the increasing frequency of CPM in the treatment of breast cancer, the oncologic benefit of this procedure is controversial in patients who do not have a genetic predisposition in developing breast cancer. Although CPM does reduce the risk of developing a contralateral breast cancer significantly, the incidence of contralateral cancers is low and has been declining over time due to advances in adjuvant chemotherapy and endocrine therapy (54). Currently, the incidence of contralateral breast cancer in patients can be estimated based on large retrospective cohort reviews and ranges from 0.3 to 1% per year depending on the age of diagnosis and characteristics of the primary tumor (54–56). The data on survival benefit of CPM are contradictory. Retrospective studies comparing unilateral mastectomy with CPM have demonstrated disease specific and overall survival benefit (57, 58). However, more recent data suggest that there is no difference in survival when breast conservation is compared with CPM (59). A recent meta-analysis conducted by Cochran Collaboration concluded that there was insufficient evidence to demonstrate a survival benefit with CPM (60).

Data on patients’ motivations for choosing CPM indicate that the patient’s choice for CPM appears to be dominated by a fear of developing another breast cancer, whereas the risk of a contralateral breast cancer and disease-specific death is routinely overestimated by patients (61–64). In a prospective survey of newly diagnosed breast cancer patients, Abbott et al. found that the mean estimated risk by patients for developing a contralateral cancer was 31% over 10 years, about ninefold the expected risk of most breast cancer patients. The perceived risk was not associated with stage, family history of breast cancer, or age of diagnosis (61). Similarly, in a qualitative study consisting of interviews with mastectomy and CPM patients, Covelli et al. noted that patients estimated a high, almost inevitable, risk of cancer recurrence and contralateral breast cancer development that they translated into a high risk of breast cancer-related death. Patients who chose CPM feared developing a contralateral cancer and the prospect of undergoing breast cancer treatment again at some point in the future (64). These results are similar to survey studies demonstrating that the most common reasons women report for choosing CPM are to avoid the development of a contralateral cancer and to improve their survival (61, 63). Other common reasons women choose CPM in these surveys were to achieve a symmetric cosmetic result, to avoid future tests and breast cancer surveillance, and to allay concern that future screening would not identify a new cancer. Given the apparent discordance between patients’ anticipated benefits of CPM and the expected oncologic benefit expected, many clinicians have called for improving communication practices and patient education in this area. Currently, the use of decision aids is being investigated as a tool to help clinicians and patients navigate decision-making in CPM (63, 65).

Although breast cancers secondary to a hereditary syndrome are uncommon, it is important to recognize that there is a population of women who do have a high risk of developing a contralateral cancer and therefore may benefit from CPM. Women with a deleterious BRCA mutation can have up to a 40% risk of developing a contralateral breast cancer over 10 years (66–68). CPM may also provide a survival benefit in deleterious BRCA mutation carriers (66, 69). Furthermore, patients with a strong family history without an identifiable genetic mutation appear to be at increased risk of developing a contralateral cancer, depending on age of diagnosis, whether the relative had a bilateral or unilateral cancer, and the degree of relative with breast cancer (first or second degree relative) (70). Genetic testing in breast cancer has also expanded to include next generation cancer panels in addition to testing for BRCA mutations. Panel testing may be appropriate for women with a strong family history without a BRCA mutation or those who have a family history indicative for more than one hereditary cancer syndrome. Unfortunately, the addition of expanded genetic testing is not without risk. Patients are more likely to test positive for a genetic variant of uncertain significance, which can make the decision-making process about prophylactic surgery even more confusing (71). Additionally, data on risk stratification for other mutations are often not as mature as the BRCA data on cancer risk, and thus even in the setting of a deleterious mutation, it is difficult to quote accurate risk to the patient. Therefore, it is important for women undergoing genetic testing to also be formally counseled on the significance of the results by a specialist trained in genetic counseling.

## Conclusion

Choosing between mastectomy and BCS can be a difficult decision involving personal preferences about body image and sexuality. In addition, external factors can influence this choice, including socioeconomic status and access to adjuvant radiation therapy, surveillance imaging, and reconstructive surgeons. Although national rates of BCS for early stage breast cancers are on the rise, rates of mastectomy have increased in young patients for reasons that are unclear. Furthermore, bilateral mastectomy has also become a common procedure in the treatment of a unilateral cancer. Most breast cancer patients are at a very low risk for developing a contralateral cancer, and yet the choice for CPM appears to be motivated by fear of developing a new cancer in the healthy breast. Although significant scientific and societal advances have been made in improving women’s choices for the breast cancer treatment, there are still deficits in the decision-making processes surrounding the surgical treatment of breast cancer. Further research is needed to define optimal patient education and shared decision-making practices in this area.

## Author Contributions

Both authors contributed to the writing and final approval of the manuscript.

## Conflict of Interest Statement

The authors declare that the research was conducted in the absence of any commercial or financial relationships that could be construed as a potential conflict of interest.
